# Supplementary material for: Health status of the red-billed tropicbird (Phaethon aethereus) determined by hematology, biochemistry, blood gases, and physical examination
Source: PeerJ. 2023 Aug 7;11:e15713. doi: 10.7717/peerj.15713 (PMC10414024; doi:10.7717/peerj.15713)
Supplement: Supplemental Information 2 [file peerj-11-15713-s002.pdf]

From: Carlos F. Mena, PhD, Director USFQ Galapagos Campus; Co-Director, Galapagos Science Center, Professor, School of Biological and Environmental Sciences.

To: Carlos Valle, PhD; Dean and Professor, School of Biological and Environmental Sciences.

Re: Manuscript “Health status of the red-billed tropic bird (Phaethon aethereus) determined by hematology, biochemistry, blood gases, and physical examination” submitted to PeerJ

Date: September 30th, 2022

Dear Dr. Valle,

This letter is to confirm that the study “Health status of the red-billed tropic bird (Phaethon aethereus) determined by hematology, biochemistry, blood gases, and physical examination” was developed under an Ecuadorian Government Research Permits, given by the Galapagos National Park, numbers PC-57-16 and PC-59-17, to C.A. Valle. The official permit regulates access, manipulation, and wellbeing of wildlife. In this specific case, of red-billed tropic bird (Phaethon aethereus) species. All protocols related to the manipulation of wildlife were in absolute compliance with stablished protocols.

I am ready to respond any further questions,

Sincerely,

Carlos F. Mena, PhD

Professor, School of Biological and Environmental Sciences.
Co-Director, Galapagos Science Center
Director, USFQ Institute of Geography
Director, USFQ Campus Galapagos
Universidad San Francisco de Quito USFQ

Avenida Alsacio Northia
Frente a Playa Mann
Puerto Baquerizo Moreno
Ecuador
(+593) 5 252 0092
info@galapagosscience.org
www.galapagosscience.org
